# Catalytic upgrading of butyric acid towards fine chemicals and biofuels

**DOI:** 10.1093/femsle/fnw064

**Published:** 2016-03-17

**Authors:** Magnus Sjöblom, Leonidas Matsakas, Paul Christakopoulos, Ulrika Rova

**Affiliations:** Biochemical Process Engineering, Division of Chemical Engineering, Department of Civil, Environmental and Natural Resources Engineering, Luleå University of Technology, SE-971 87 Luleå, Sweden

**Keywords:** butyric acid, butanol, catalytic upgrade, butyl-butyrate, esterification, lipase

## Abstract

Fermentation-based production of butyric acid is robust and efficient. Modern catalytic technologies make it possible to convert butyric acid to important fine chemicals and biofuels. Here, current chemocatalytic and biocatalytic conversion methods are reviewed with a focus on upgrading butyric acid to 1-butanol or butyl-butyrate. Supported Ruthenium- and Platinum-based catalyst and lipase exhibit important activities which can pave the way for more sustainable process concepts for the production of green fuels and chemicals.

## Abstract (precis)

Novel chemical and biochemical catalytic methods for upgrading carboxylic acids to fine chemicals and biofuels are reviewed.

## INTRODUCTION

**Figure 1.**
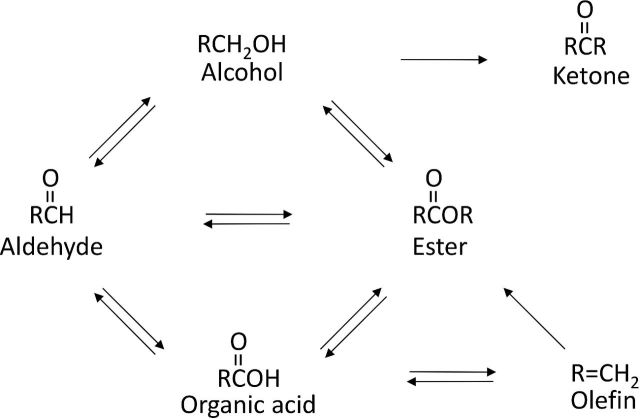
Different upgrading pathways from organic acids. Adapted from Eggeman and Verser (2005).

From the perspective of a biobased economy, it is imperative to utilize renewable resources and to find efficient and innovative value chains from biomass to commodity chemicals and biofuels (Koutinas *et al*. 2014; Straathof 2014). Carboxylic acids are an important group of organic acids which are extensively used in the food and chemical industry. Fermentative production of organic acids can be very efficient and several, including citric acid, lactic acid, itaconic acid, succinic acid etc., are being produced on a commercial scale (Lópes-Garzón and Straathof 2014). In principle, the functionality of carboxylic acids facilitates catalytic upgrading to broad range of compounds including aldehydes, esters, ketones, alcohols, alkanes, alkenes and olefins (Fig. 1) (Eggeman and Verser 2005). Fermentative production of organic acids is generally more efficient in comparison with the production of their corresponding aldehyde, alcohol, alkene or alkane. For example, the maximum concentration of 1-butanol obtained during fermentation is usually between 10 and 20 g L^−1^ whereas that of butyric acid is close to 90 g L^−1^ (Straathof 2014). Similarly, the obtainable concentration of propionic acid is about 105 g L^−1^ whereas it is 10.8 g L^−1^ for 1-propanol (Straathof 2014). Other groups of compounds such as esters and ketones are also very interesting target products which can be derived from carboxylic acids and used as commodity chemicals and biofuels (Gaertner *et al*. 2009; Ju *et al*. 2010; Chuck and Donnelly 2014). Hence, there is a great interest to explore methods to upgrade carboxylic acids to its derivatives as means for more efficient process concepts. This paper will review current state of the art technologies for the catalytic upgrading of butyric acid with particular focus on 1-butanol or butyl-butyrate as the target products. Both chemocatalytic and biocatalytic strategies will be covered.

## CURRENT STATUS OF BUTYRIC ACID PRODUCTION

Butyric acid is an ill-smelling 4-carbon carboxylic acid which has important applications in the solvent, polymer and specialty chemical market and is currently produced by petrochemical means through the oxy process (Mascal 2012). Microbial production of butyric acid production is performed anaerobically and is generally robust and straight forward with acetic acid being the most notable side product (Fayolle, Marchal and Ballerini 1990). *Clostridium tyrobutyricum* has been identified as the most efficient producer of butyrate, reaching concentrations up to about 60 g L^−1^ in batch fermentation and up to about 73 g L^−1^ in fed-batch cultivation using glucose as the carbon/energy source with the corresponding productivities of 0.78 and 1.41 g L^−1^·h, respectively (Song *et al*. 2010). Using *C. tyrobutyricum* immobilized on fibrous beds and a repeated fed-batch technique a butyric acid concentration of 87 g L^−1^ could be reached on glucose with a productivity of 1.1 g L^−1^·h (Jiang *et al*. 2011). Continuous production with cell recycle has been used to reach a productivity of 9.5 g L^−1^·h while maintaining a butyrate concentration of 29.7 g L^−1^ (Michel-Savin, Marchal and van de Casteele 1990). *Clostridium tyrobutyricum* can utilize several sugars including glucose, fructose, xylose and arabinose (although some of them may not be co-utilized) and is hence well suited to ferment lignocellulosic hydrolysates (Zhu, Wu and Yang 2002; Huang *et al*. 2011). For example, using a fed-batch process and a combination of sweet sorghum stalk hydrolysate and beet molasses as feed-stock a butyric acid concentration of 58.8 g L^−1^ was achieved with corresponding yield and productivity 0.52 g g^−1^ fermented sugars and 1.9 g L^−1^·h, respectively (Sjöblom *et al*. 2015).

## RECOVERY OF BUTYRIC ACID

Recovery costs are generally in the range of 30%–40% of the total production cost for carboxylic acids, and it is hence crucial to find competitive process concepts in order to make biobased production economically feasible (Lópes-Garzón and Straathof 2014). Product recovery of carboxylic acids from fermentation broths is challenging as they are highly water soluble, can shift between the carboxylate and acid form and are often diluted. Extraction and adsorption are the most prevalent recovery methods although the requirement to completely convert the carboxylate into its acid or carboxylate form before recovery may consume substantial amounts of mineral acids or bases (Lópes-Garzón and Straathof 2014).

Considering biobutanol or butyl-butyrate production from butyric acid, reactive extraction and reactive regeneration by esterification has received attention as they can minimize waste and material use (Eggeman and Verser 2005). In a patent application (Kang *et al*. 2011), a method was described where butyric acid was complexed with tripentylamine forming a water insoluble phase which subsequently was decomposed in a distillation column enabling reuse of the amine and recovery of pure butyric acid in the top. Extraction trials made with tripentylamine showed that 99% of the butyric acid could be recovered in the amine layer.

Aqueous two-phase partition is a liquid–liquid extraction method which was evaluated for the separation between butyric acid and acetic acid in fermentation broth (Wu *et al*. 2010). Exploiting the tendency for acetic acid to remain in the aqueous salt solution, butyric acid could selectively be salted out of the aqueous solution by the addition of calcium chloride causing two phases to appear where the upper phase is enriched in the butyric acid. With this method, the ratio BA/AA could be increased from 4.02 to 8.9 in the upper phase of the fermentation broth. Extraction of butyric acid from *C. tyrobutyricum* fermentation broths were also studied in a polyethylene glycol/Na_2_SO_4_ aqueous two-phase system (Wu *et al*. 2015). In this method, Na_2_SO_4_ was first added to salt out cell protein, sugars and nitrogen compounds, followed by filtration and transferring to PEG. The major components of the resulting PEG-rich phase were butyric acid, acetic acid and butanol. The PEG was precipitated with an iodine solution and filtered. The filtrate was subsequently distilled to separate butyric acid, where at the optimal conditions a yield as high as 91.74% ± 0.46% was demonstrated.

Zeolites could be a possible option for recovery of butyric acid from fermentation broths by adsorption. When a hydrophobic MFI type zeolite was investigated for the selective adsorption of 1-butanol from model ABE fermentation broths, butyric acid was co-adsorbed with 1-butanol (Faisal *et al*. 2014). Without butanol present, selective adsorption of butyric acid could be accomplished. The results obtained are encouraging; however, more experiments should be performed using real fermentation broths and column breakthrough experiments.

## CHEMOCATALYTIC UPGRADING OF CARBOXYLIC ACIDS

The upgrading of carboxylic acids to their corresponding aldehydes, alcohols and hydrocarbons require carefully balanced oxygen removal reactions. Several catalytic routes can be applied for this purpose including dehydration, hydrogenolysis and hydrogenation (De, Saha and Luque 2015). In order to produce larger molecules appropriate for use as diesel and jet fuels, C–C coupling reactions such as ketonization or esterification reactions can be exploited (Gaertner *et al*. 2009; Ju *et al*. 2011; Chuck and Donnelly 2014). In the literature, there are several patents and reports describing the preparation and application of mixed heterogeneous metal catalysts on various supports (for example, RuCoPdZn/Al_2_O_3_, Co-Pt/SiO_2_ and Ru-Sn/Al_2_O_3_) for the hydrogenation of C1-C6 carboxylic acids to produce biofuels (Pesa, Graham and Kliewer 1984; Yang 2008; Johnston *et al*. 2009). By carefully adjusting the reaction conditions and the composition of the catalysts, the activity and selectivity can be adjusted to favor the aldehyde, ester or alcohol. For example, whereas Ru/TiO_2_ showed 98% selectivity toward ethylacetate, Pt-Sn/TiO_2_ displayed 99% selectivity for ethanol during catalytic hydrogenation of acetate (Mclachlan, Pimblett and Price 1991).

Development of a catalyst with the proper activity, selectivity and stability is challenging, particularly considering the corrosive nature of monocarboxylic acids and the low reactivity of the carboxyl group in comparison to other carbonyl groups such as ketones and aldehydes (Manyar *et al*. 2010; Lee *et al*. 2014). Catalyst poisoning, oxidation of the metal, loss of metal due to leaching, loss of metal specific surface area due to sintering or pore occlusion and coke formation must be minimized to prevent deactivation of the catalyst (Santillan-Jimenez and Crocker 2012). In order for chemocatalytic upgrading to be commercially applicable, efficient recovery processes of the carboxylic acid in combination with cost effective catalytic systems must be developed. Integrated recovery and upgrading systems are highly attractive as they can minimize waste and energy consumption.

For the conversion of acetic-, hexanoic-, nonanoic- and dodecanoic acid to their corresponding aldehydes, the oxide catalysts Fe_2_O_3_, Cr_2_O_3_, Cr_2_O_3_/TiO_2_ and V_2_O_5_/TiO_2_ have shown high selectivity. Ru-Sn/Al_2_O_3_ the conversion of acetic acid was 85% with a selectivity of 59% for the aldehyde at 300°C (Yokoyama and Yamagata 2001).

Reactive extraction of butyric acid with a trialkylamine followed by esterification and hydrogenolysis was previously mentioned as an interesting route for biobased production of butyl-butyrate or 1-butanol from butyric acid. In principle, 1 mol of butyric acid and 1 mol of 1-butanol are esterified to 1 mol of butyl-butyrate which subsequently is hydrogenolyzed to 2 mol of 1-butanol (Ju *et al*. 2010; Kang *et al*. 2011). One part of the butanol is then used as a product and one part is used for the esterification reaction. The intermediate step of esterification allows milder conditions to be used compared to direct catalytic conversion to butanol, although the development of new catalysts and process design may level this difference. The hydrogen produced by *C. tyrobutyricum* could in principle be used for the hydrogenolysis reaction making the process more sustainable (Kang *et al*. 2011).

Esterification trials between butyric acid and 1-butanol have been made using various catalysts including the commercially available strong ion exchange resin catalyst DOWEX 50WX8-400, DOWEX 50WX2-400, Amberlyst 70 and Amberlyst 121 at different temperatures (100°C –120°C) and different butanol to butyric acid molar ratios (2 and 3). Conversion, selectivity and yield of butyl-butyrate were found to be high, typically ranging between 90% and 99% (Table 1) (Kang *et al*. 2011). The stability was reported to be good with a conversion and selectivity of 99% and 97%, respectively, over 1 month with Amberlyst 70 and a reaction temperature of 110°C.

**Table 1.** Summary of data for chemocatalytic upgrading of butyric acid to various derivatives.

		Target	T	Press	Conversion	Selectivity for	Yield	
Catalyst	Reaction	product	(°C)	(atm)	(%)	target prd (%)	(%)	Reference
Dowex 50Wx8-400	Esterification of BA and ButOH	BuB	100	na	97.3	99.4	96.7	Kang *et al*. (2011)
Dowex 50Wx2-400	Esterification of BA and ButOH	BuB	120	na	97.5	98.9	96.4	Kang *et al*. (2011)
Amberlyst 70	Esterification of BA and ButOH	BuB	110	na	98.2	98.0	96	Kang *et al*. (2011)
Amberlyst 121	Esterification of BA and ButOH	BuB	110	na	96.2	99.1	95.3	Kang *et al*. (2011)
1%Pa-Cu/ZnO/Al_2_O_3_	Hydrogenolysis of BuB	ButOH	175	10	98.7	99.8	98.5	Kang *et al*. (2011)
Ru/ZnO	Hydrogenation of BA	ButOH	265	25	62.4	97.8	Na	Lee *et al*. (2014)
1Ru-2Sn/ZnO	Hydrogenation of BA	ButOH	265	25	99.9	98.6	Na	Lee *et al*. (2014)
1.0Ru/C	APHDO of BA	ButOH	190	63	93.5	3.3	Na	Chen *et al*. (2011)
MnO_x_/CeO_2_	Ketonization of BA	4HN	410	1	28	>98	Na	Murkete, Jackson and Miller (2011)
CM-HMS	Ketonization of BA	4HN	410	1	36	>98	Na	Murkete, Jackson and Miller (2011)
CM-MCM-41	Ketonization of BA	4HN	410	1	31	>98	Na	Murkete, Jackson and Miller (2011)

APHDO = aqueous phase hydrodeoxygenation; BA = butyric acid; BuB = butyl butyrate; ButOH = 1-butanol; CM = Ce + Mn; 4HN = 4-heptanone; HMS and MCM-41 are mesoporous silica; Na = not available.

Subsequent hydrogenolysis of butyl-butyrate to butanol was tested with a commercially available Cu/ZnO/Al_2_O_3_ catalyst prepared with 1.0 wt% palladium. With a downstream reactor pressure of 10 atm, the hydrogenolysis reaction was favored when the temperature increased from 150°C to 200°C, the hydrogen to butyl-butyrate ratio increased from 10 to 35 and the space velocity was decreased from 1 to 0.3 h^−1^. The yield for the hydrogenolysis reaction was decreased from about 92% to 86% over 720 h.

There are also promising catalysts for direct conversion of butyric acid to 1-butanol using ZnO supported Ruthenium–Tin bimetallic catalysts (Lee *et al*. 2014). At 265°C and a hydrogen pressure of 25atm, vapor phase hydrogenation showed 99.9% conversion of butyric acid with a selectivity of 98.6% for 1-butanol. Lasting for 145 days without significant deactivation, the 1Ru-2Sn/ZnO catalyst showed a very high stability and durability. The direct conversion could facilitate a simpler process design and a better economy compared intermediate esterification.

Liquid phase hydrogenation using titania supported Pt and Pt-Re bimetallic catalysts were evaluated on a selection of aliphatic and cyclic carboxylic acids (Manyar *et al*. 2010). These catalysts exhibited high activity at relatively mild conditions (130°C and a hydrogen pressure of 19.7atm). Generally, it was observed that the Pt-Re/TiO_2_ catalysts had reduced selectivity toward the alcohol compared to the Pt/TiO_2_ catalysts. Aqueous phase hydrogenation or hydrodeoxygentation (APHDO) is very important reactions because they would allow the catalytic upgrading to occur in water solutions, which is the intrinsic solvent of carboxylic acids produced by fermentation, hence, reducing energy intensive and costly water removal operations (Chen *et al*. 2011). Carbon supported Ruthenium (Ru/C) catalysts that have shown promising results for the aqueous phase hydrogenation of lactic acid to propylene glycol performed at 100°C –170°C and hydrogen pressures of 7–14MPa, displaying selecitivies of 90%–95% and nearly complete conversion (Zhang, Jackson and Miller 2001).

APHDO of mono carboxylic acids in order to produce their corresponding alcohols or alkanes was investigated using Ru/C, Ru/ZrO_2_ and Ru/Al_2_O_3_ catalysts at a hydrogen pressure of 6.4 MPa (63.2 atm) (Chen *et al*. 2011). With C2–C4 carboxylic acids and 1.0Ru/C catalysts, it was generally found that when the reaction temperature increased from 150°C –190°C the conversion increased at the expense of the selectivity for the alcohol. At the high temperature, the C–C bond cleavage reaction was dominant and the major products were alkanes with one less carbon than the original acid. For butyric acid, the conversion was 93.5% but the selectivity for 1-butanol was only 3.3%, with 22.3% for methane, 0.2% for ethane, 64.4% for propane and 9.6% for butane. Further, studies on propionic acid showed that the use of Al_2_O_3_ support could largely inhibit the C–C bond cleavage reaction in contrast to C or ZrO_2_ supports.

In addition to esterification, ketonization is another interesting C–C coupling reaction which could be used to create larger chain molecules appropriate for diesel and jet fuels (Gaertner *et al*. 2009). Ceria-zirconia catalysts and supported mesoporous solid base catalysts are promising for ketonization reactions (Gaertner *et al*. 2009; Murkete, Jackson and Miller 2011). The synthesis of 4-heptanon from butyric acid was investigated using supported mesoporous solid base catalysts at 410°C and atmospheric pressure (Murkete, Jackson and Miller 2011). The conversion was about 30% with a selectivity larger than 98% toward 4-heptanone in all cases.

## BIOCATALYTIC CONVERSION

### Lipase catalyzed esterification

Enzymatic catalysis is gaining increasing attention as it is considered to be more environmental friendly compared to the chemical catalysis. Normally, lower temperatures are required compared to chemical catalysis and there is no need for use of harsh chemicals, preventing also the generation of wastes that need to be further treated. Moreover, enzymatic reactions are very selective, minimizing the formation of by-products. Esterification of organic molecules (e.g. butanol) can yield esters, like butyl-butyrate, with excellent characteristics as fuel additives. These esterification reactions are carried out by lipases (triacylglycerol acylhydrolase; EC 3.1.1.3). Although the mode of action of lipases involves the hydrolysis of ester bonds they differ from the other esterases due to the fact that they demonstrate a unique action in the interface of aqueous and organic solutions (Pandey *et al*. 1999). Lipases are catalyzing both hydrolysis and esterification reactions depending on the reaction conditions.

Lipases have the ability to perform esterification between a wide variety of alcohols (acceptor) and organic acids (donor). For example, Martins *et al*. (2014) evaluated the esterification efficiency of different commercial lipases (Novozym 435, Lipozyme RM-IM and Lipozyme TL-IM) on a variety of alcohols (ethanol, isopropyl alcohol, butanol and pentanol) and organic acids (acetic, propionic and butyric acid). Butanol was an excellent candidate as it was efficiently esterified with all the acids.

Novozym 435 (*Candida antartica* lipase B) was used for the esterification of 1-butanol with acetic acid and the formation of butyl-acetate by Martins *et al*. (2011), where a high conversion rate (91.5%) was achieved (Table 2). Moreover, the authors demonstrated that the lipase can be re-used for several cycles without significant loss in activity if a washing step with solvent takes place between batches. This positive effect of ‘solvent-washing’ step was also demonstrated in another work of Rodrigues *et al*. (2008). Re-use of the lipase is of great importance for industrial applications, as it reduces the operational costs. In another work with the same enzyme, Martins *et al*. (2013c) examined the effect of low-frequency ultrasound (40 kHz) on the efficiency of butyl-acetate formation. The authors demonstrated that the ultrasound improved the process not only by increasing the productivity (7.5-fold higher compared to the control), but also by improving the stability of the enzyme in higher amounts of acetic acid (2M instead of 0.3M) and also by enabling the direct re-use of the lipase. Ultrasounds (40 kHz, 220W ultrasonic bath) were also used by Paludo *et al*. (2015). Moreover, they demonstrated that the use of molecular sieves during esterification can provide a water-free environment, as it removes the water released during the esterification, and resulted in re-using the lipase for ten cycles without any loss of activity. Another enzyme that was used for the preparation of butyl-acetate is Lipozyme IM20 (lipase from *Rhizomucor miehei*) (Kumar and Rao 2003). Among the different solvents evaluated, *n*-hexane, isooctane, cyclohexane and *n-*heptane were the most efficient, whereas chloroform, dioxane and THF resulted in very poor esterification due to the destruction of the essential hydration layer and subsequently deactivating the lipase.

**Table 2.** Summary of data for enzymatic upgrading of butyric acid and butanol to various derivatives.

Product	Donor	Acceptor	Lipase	System	Yield (Time)	T (°C)	Reference
Butyl-acetate	Acetic acid	Butanol	Novozym 435	Hexane	91.5% (2.5 h)	40	Martins *et al*. (2011)
Butyl-acetate	Acetic acid	Butanol	Novozym 435	Hexane	94% (2.5 h)	46	Martins *et al*. (2013a)
Butyl-acetate	Acetic acid	Butanol	Lipozyme IM20	Hexane	90% (96 h)	45	Kumar and Rao (2003)
Butyl-butyrate	Butyric acid	Butanol	Type VII	Heptane	75% (24 h)	41	Santos and de Castro (2006)
Butyl-butyrate	Butyric acid	Butanol	Lipozyme RM-IM	Hexane	>90% (16 h)	40	Lorenzoni *et al*. (2012)
					94.8% (24 h)		
Butyl-butyrate	Butyric acid	Butanol	Lipozyme TL-IM or MCI-TLL	Hexane	∼95% (7 h^a^)	50	Martins *et al*. (2013b)
Butyl-butyrate	Butyric acid	Butanol	Lipozyme TL-IM	Hexane	95% (3 h)	48	Martins *et al*. (2013c)
Butyl-butyrate	Butyric acid	Butanol	*Mucor* sp. lipase	Cyclohexan	95% (24 h)	35	Abbas and Comeau (2003)
Butyl-caproate	Caproic acid	Butanol	*Mucor* sp. lipase	Cyclohexan	100% (24 h)	35	Abbas and Comeau (2003)
Ethyl-butyrate	Butyric acid	Ethanol	Lipozyme TL-IM	Solvent-free	90% (6 h)	30	Paludo *et al*. (2015)
Butyl-butyrate	Butyric acid	Butanol	Novozym 435	Hexadecane in aqueous broth media	34 mM	37	van den Berg *et al*. (2012)

a The time was taken by a figure of the article.

Santos and de Castro (2006) used a lipase from *C. rugosa* (lipase type VII) for the formation of butyl-butyrate esters resulting in a concentration of 32.4 g L^−1^. Another lipase from *R. miehei* (Lipozyme RM-IM) was used for the same esterification by Lorenzoni *et al*. (2012). This lipase presented high efficiency toward butyl-butyrate resulting in a concentration of 948 mM (24 h) with a productivity of 1.22 and 0.85 mmol of ester/(g of catalyst)ċh after 16 and 24 h, respectively. Another commercial lipase that was used for the preparation of butyl-butyrate was Lipozyme TL-IM (from *Thermomyces lanuginosus* lipase—TLL). Martins *et al*. (2013a) compared an *in house* immobilized TLL with MCI GEL CHP20P (MCI-TLL) and an already immobilized TLL in silicate support (Lipozyme TL-IM). Although with both support materials the TLL resulted in the same conversion (around 95%), the initial conversion rates of MCI-TLL were higher and the productivity of MCI-TLL was 14.5 mmol g^−1^·h compared to 3.2 mmol g^−1^·h of the Lipozyme TL-IM. This difference in productivity was a result of the ability of MCI-TLL to act with higher concentrations of butyric acid, underpinning the importance of appropriate selection of supporting material for immobilization. The same lipase was efficiently used by another work, demonstrating high re-usability when washed with *n-*hexane (Martins *et al*. 2013b). Finally, a non-commercial lipase from *Mucor* sp. was also evaluated in a combinations of acids (propionic, butyric and caproic) and alcohols (methanol, ethanol, allyl, butanol, isoamyl, geraniol, citronellol and farnesol) resulting in high yield of esters (Abbas and Comeau 2003).

All the aforementioned works performed the esterification in ‘pure’ 1-butanol. On the other hand, due to the relative low concentrations of 1-butanol in the fermentation broths of ABE, there is a great interest to enable the direct esterification of 1-butanol and butyric acid *in situ*, avoiding energy demanding downstream processes of separation. By applying this process not only the cost of the process is reduced, but also the levels of 1-butanol are kept low minimizing the inhibitory effect toward the fermenting bacteria. A phase of an organic solvent is also necessary in order to enable the esterification to take place. When aiming for diesel enrichment with butyl-butyrate, the possibility of direct esterification in the diesel is interesting as there would be no need to separate the ester after its synthesis, hence, leading to more practical and economical systems for the production of ‘green’ fuels. An interesting feature about lipases is that they catalyze the ester synthesis particularly well in inorganic solvents including *n-*alkanes such as hexane, dodecane and hexadecane, which are major constituents of petroleum fuels (Zaks and Klibanov 1985). The fact that the enzyme catalyzed synthesis of butyl-butyrate could be performed in these types of hydrocarbons opens up the possibility for one-pot diesel enrichment with butyl-butyrate. This process was evaluated by van den Berg *et al*. (2012) by using Novozym 435 to directly esterify 1-butanol and butyric acid that are produced during ABE fermentation in a hexadecane phase. The highest concentration that was detected in the hexadecane phase was 4.9 g L^−1^ (34 mM), which although is not very high, and it is a first step for the development of an efficient one-pot process. Preliminary work by the authors have demonstrated that esterification of 1-butanol and butyric acid to butyl-butyrate could be performed directly in diesel fuel with a conversion efficiency of 70%–80% using Novozym 435 (work in progress).

### Whole-cell hydrogenation of butyric acid

Another approach of biological conversion of butyric acid to 1-butanol involves the whole-cell catalysis with the hyperthermophile *Pyrococcus furiosus*, which has the ability to hydrogenate carboxylic acids selectively to the corresponding alcohols (Ni *et al*. 2012). More specifically, the authors mentioned that *P. furiosus* has the ability to couple H_2_ oxidation with the reduction of carboxylic acids under highly chemoselective conditions. Although the conversion of butyric acid to 1-butanol was only 28% (compared to, e.g. pentanoic acid to *n-*pentanol which accounted for >99%), it was an important discovery and further improvements can be made.

### Microbial electro synthesis of 1-butanol

The ability to increase the reduction potential of microorganisms having butanol as an intermediate in their metabolic pathways using electricity has shown that it possible to produce 1-butanol from butyric acid or glycerol (Sharma *et al*. 2013; Choi *et al*. 2014). Hence, coupling electricity derived from wind-, hydro- and solar power, with microbial reduction of various carbon sources could be an efficient way of storing excess electrical energy in the form of biofuel molecules and organic commodities.

## CONCLUSIONS

The research in catalytic upgrading of carboxylic acids has made a very important progress during the last years. Upgrade can be performed with both chemical and biological routes. Ru-Sn/Al_2_O_3_ catalyts may be a good starting point for upgrading of butyric acid to butanal through hydrogentation. For direct catalytic conversion of butyric acid to 1-butanol 1Ru-2Sn/ZnO and Pt/TiO_2_ catalysts are promising. For APHDO, Ru/Al_2_O_3_ appear promising for conversion of BA to 1-butanol. For esterification of BA and 1-butanol DOWEX 50WX8-400, DOWEX 50WX2-400, Amberlyst 70 and Amberlyst 121 have shown good results. Ketonization to 4-heptanone may be performed with ceria-zirkonia or supported mesoporous solid base catalysts. On the other hand, biological catalysis can take place with either enzymes or whole cells. Different enzymes have been used, which resulted in high conversion rates. One important aspect of enzyme use is the ability to re-cycle and re-use the catalyst for several catalysis cycles. This was demonstrated by either applying a solvent washing step or by using ultrasounds. On the other hand, whole-cell catalysis is a ‘technology’ not fully explored and its maximum potential needs to be discovered.
